# Medication-related osteonecrosis of the jaw (MRONJ) and eNOS Polymorphisms in multiple myeloma patients: a single center experience

**DOI:** 10.1186/s12903-021-01634-9

**Published:** 2021-05-18

**Authors:** Betul Taş Ozyurtseven, Istemi Serin, Ayse Feyda Nursal, Sacide Pehlivan, Mustafa Pehlivan

**Affiliations:** 1 Department of Oral and Maxillofacial Surgery, Faculty of Dentistry, Gaziantep University, Gaziantep, Turkey; 2 Department of Medical Genetics, Faculty of Medicine, Hitit University, Corum, Turkey; 3 Department of Hematology, Faculty of Medicine, Gaziantep University, Gaziantep, Turkey; 4 Department of Medical Biology, Istanbul Faculty of Medicine, Istanbul University, Istanbul, Turkey; 5 Department of Hematology, Istanbul Training and Research Hospital, University of Health Sciences, Org. Nafiz GURMAN Cad. 34098 Fatih, Istanbul, Turkey

**Keywords:** Medication-related osteonecrosis of the jaws (MRONJ), Bisphosphonate, Multiple myeloma, Endothelial nitric oxide synthase

## Abstract

**Background:**

Multiple myeloma (MM) constitutes approximately 10% of hematological malignancies. Bisphosphonates have established themselves in solid organ metastasis and multiple myeloma lytic bone lesions by inhibiting osteoclast activation. Medication-related osteonecrosis of the jaw (MRONJ) emerges as an important complication. Investigating host-based factors, and developing personal risk factors gain importance in the development mechanism of MRONJ. We aimed to reveal the different genotype polymorphisms, and clinical effects of eNOS in patients with a diagnosis of MRONJ in MM patients.

**Methods:**

Medical records and blood samples were collected from 60 MRONJ patients with MM and 60 healthy controls. Inclusion criteria was having an exposed maxillofacial bone for more than eight weeks, a history of bisphosphonates, and no history of radiation therapy for the jaws. eNOS *G894T* and *intron 4 VNTR* were calculated by polymerase chain reaction and/or restriction fragment length polymorphism.

**Results:**

eNOS *G894T* and *VNTR* genotypes and alleles were compared statistically with the healthy control group. There was no significant difference between the two groups. In comparison between *G894T* and clinical parameters, aphthous stomatitis was more common in TT genotype, while DMFT > 3 was more common in TG-GG genotype (*p* = 0.035, 0.023).

**Conclusions:**

eNOS induces osteogenesis in bone metabolism, with its regulatory effects on bone remodeling and also NO induced angiogenesis takes place indirectly with its protective effect on endothelial functions. We see that these polymorphisms affecting the entire process of bone remodeling and angiogenesis, especially mucosal damage, which is the triggering factor of MRONJ pathology, have been revealed in the MM patient group. Considering the MRONJ initiating factors, it is necessary to emphasize the importance of our study results. It should be seen as an important step for new studies towards MRONJ and its treatment.

## Background

Multiple myeloma (MM) constitutes approximately 10% of hematological malignancies. It is a disease caused by malignant plasma cells, the pathology of abnormal proliferation, causing free light chain release accompanying the increase in monoclonal immunoglobulin [1]. The data obtained from many publications indicated that MM affects 7 in every 100,000 adults and the median age is 65 years old [2–4]. Clinical manifestations appear as renal failure, anemia, hypercalcemia and lytic bone lesions. Although positron emission tomography/computed tomography (PET/CT) is often preferred for the detection of lytic bone lesions, magnetic resonance (MR), computed tomography (CT), and direct radiographs also contribute to the diagnosis.

Metastatic bone lesions can be encountered in many solid malignancies. In this patient group, cancer cells that metastasize to bone stimulate osteoclasts via the cytokines they produce and cause bone destruction [5]. Lytic bone lesions in multiple myeloma occur by a similar mechanism. They cause bone destruction by secreting osteoclast stimulants such as nuclear-KB ligand (RANKL) receptor activator as well as molecules that inhibit osteoblastic activity such as dickkopf-1 (DKK) [5, 6]. Bisphosphonates have established themselves in solid organ metastasis, and multiple myeloma lytic bone lesions by inhibiting osteoclast activation [5–7]. Decreased osteoclast activity decreases bone remodeling and is used to prevent osteoporosis by increasing bone density [5–7].

Besides all these effects; medication-related osteonecrosis of the jaw (MRONJ) emerges as an important complication [8]. Although the exact mechanism of medication-related osteonecrosis has not yet been determined, several potential hypotheses have been proposed. The most accepted hypothesis is that deep inhibition of osteoclast function inhibits normal bone turnover and damage from normal mechanical load or any injury [5, 8]. In addition, studies with different mechanisms can be also mentioned. In a review about non-bisphosphonate related osteonecrosis of jaw, authors divided reasons related to osteonecrosis following mechanisms: Systemic medication, radiation, bacterial, viral or deep fungal infections, direct chemical toxicity, trauma or idiopathic [9]. In reports involving non-bisphosphonate related osteonecrosis cases, we see that the main reasons were trauma, local interventions, chemotherapy and systemic steroid usage [10, 11]. With all these findings, it can be mentioned that the responsibility of MRONJ is related to more than one factor or the necessity of association.

In a study related to bisphosphonates usage, it was shown that zoledronic acid exerts an inhibitory effect on circulating levels of vascular endothelial growth factor (a potent angiogenesis stimulator) [12, 13]. Bisphosphonates were also thought to be toxic to the oral mucosa directly, and it was reported that they could cause mucosal fenestration and exposure to bone [14]. MRONJ and its associated maxilla-mandibular involvement are related to the “intraoral environment”. In addition, knowing that bisphosphonates accumulate especially in bones with high turnover capacity, it should be considered that the bisphosphonate concentration of the maxilla and mandible is extremely high [5, 15]. There are also studies contrary to this concentration theory. Bauss et al. [16] have been shown that ibandronate uptake is not different in jaw compared to long bones and vertebrae. Morris et al. [17] also showed that there was no difference in mandible binding in patients with metastatic malignancies who used chronic bisphosphonates. Otto et al. [18] showed that the development of osteonecrosis increased in conditions that create an acidic local environment such as infections during high dose use of non-nitrogenous bisphosphonates (zoledronate and ibandronate).

Dentoalveolar trauma is the most common and consistent risk factor in most MRONJ cases reported to date. Patients with a history of inflammatory dental disease (e.g. periodontal and dental abscesses) are at a seven-fold increased risk for developing MRONJ [19, 20]. The duration of bisphosphonate therapy has been associated with the risk of developing both MRONJ and more severe disease [5]. Similarly, the more potent pamidronate, and especially intravenous bisphosphonates such as zoledronic acid appear to be significantly more problematic compared with oral bisphosphonate drugs [5, 21].

Investigating host-based factors and developing personal risk factors gain importance in the development mechanism of MRONJ. At this point, nitric oxide synthase (NOS) is an important individual factor. NO synthesized from L-arginine by the NOS enzyme. NOS can also catalyze superoxide anion production due to the presence of substrate and cofactor. There are three main isoforms of the enzyme called neuronal NOS (nNOS), inducible NOS (iNOS) and endothelial NOS (eNOS). These differ in their dependence on Ca2^+^, their expression and activity. NOS is an important signaling molecule in bone [22]. In addition, physiological activity appears to be source and concentration dependent [12–23]. Nitric oxide (NO) produced by osteocytes is an important regulator of bone response to movement and compression and has been shown to mediate osteoclast activity. iNOS has been shown to mediate inflammation-induced bone resorption on the compression side [24]. Although osteogenesis on the tension side is not well understood, there are publications reporting the increase and role of eNOS in bone formation. As a result, NO and NOS play an important role in bone formation and turnover.

In our study, we aimed to reveal the different genotype polymorphisms and clinical effects of eNOS in patients with a diagnosis of MRONJ, which is frequently encountered in the MM patient group and creates clinical difficulties.

## Methods

### Study population

Medical records and blood samples were collected from 60 MRONJ patients with MM (31 females, 29 males; mean age: 53 years old) and 60 healthy controls (29 females, 31 males; mean age: 54 years old). The patients diagnosed and treated in the Department of Hematology, Faculty of Medicine, Gaziantep University (Gaziantep, Turkey). Demographic characteristics were recorded, and clinical/radiographic, dental examinations were performed for the patients diagnosed with MM. Presence of MRONJ and symptoms were examined by an Oral Surgeon from Faculty of Dentistry, Gaziantep University, (Gaziantep, Turkey). Inclusion criteria were having an exposed maxillofacial bone for more than eight weeks, a history of bisphosphonates and no history of radiation therapy for the jaws.

Oral mucosal physical examination findings were analyzed under two subheadings together with MRONJ: Presence of aphthous stomatitis and calculation of the decayed-missing-filled teeth index (DMFT score). DMFT were recorded and calculated for each case. The healthy control group was similar in terms of age and sex distribution; with healthy individuals who did not have any evidence of any malignancy. All patients were of Turkish origin, and their informed consent was obtained. The study protocols were performed according to the principles of the Declaration of Helsinki. The experimental study protocol and process were assessed and approved by the Faculty of Medicine, Gaziantep University ethics committee (2018/78).

### Genotyping of eNOS gene variants

For the analysis of eNOS gene variants, Vacutainer tubes with EDTA as anticoagulant were used to collect about 5 mL peripheral blood. DNA was extracted from leukocytes according to standard protocols [25]. *G894T* and *intron 4 VNTR* were calculated by polymerase chain reaction (PCR) and/or restriction fragment length polymorphism (RFLP) as previously described [26, 27].

### Statistical analysis

The OpenEpi Info software package version 3.01 (www.openepi.com) and The Statistical Package for the Social Sciences program (IBM SPSS, version 20) were used for Statistical analysis. Results were presented as mean ± standard deviation (S.D). The χ^2^ test, analysis of variance (ANOVA) statistics, and the Fisher exact test were used to analyze the relationships between *eNOS* variants and the clinical/demographical characteristics. Risk factors were calculated using Odds Ratios (ORs) and 95% confidence intervals (CIs). A *p* value of less than 0.05 was considered significant.

## Results

Of the 60 patients included in our study, 31 were female (52%) and 29 were male (48%). Only 5 (16.6%) of the patients were diagnosed with light chain. The 5-year OS was 76%, the PFS median was 54.3 months and the median follow-up was 36 months (7.8–99.1). Demographic data can be seen in Table 1.

**Table 1:** Clinical features and treatment regimens of MRONJ patients

	MRONJ patients	Controls
	n:60 (%)	n:60 (%)
Age	53 (37–71)	54 (29–71)
Gender		
Females/males	31/29 (52/48)	29/31 (48/52)
Ig subtypes		
κ/λ	22/11 (64.9/35.1)	
G/A	23/5 (67.4/16)	
Light chain	5 (16.6)	
Stage (Salmon–Durie)		
II/III	10/23 (31/69)	
A/B	27/6 (76/24)	
IPI		
I	10 (30.2)	
II/III	9/13 (26.6/43.2)	
ECOG		
> 1	4/33 (16.6)	
Hemoglobin		
gr/dL	10.7 (6.6–14.7)	
Leucocytes		
μL	6100(2760–17,300)	
Platelets		
10^3^/μL	198 (123–337)	
C-reactive protein		
mg/dL	6.6 (2.1–215)	
LDH		
IU/L	235 (100–556)	
β2-microglobulin		
mg/L	4.7 (1.5–47)	
Albumin		
g/L	3.6 (2.2–5.1)	
Treatment		
VCD, ASCT, LD	60	
OS % (5 years)	76	
PFS (month)^a^	54.3	
Follow up duration^a^ (month)	36 (7.8–99.1)	

VCD, bortezomib, cyclophosphamide, dexamethasone; APBSCT: autologous peripheral blood stem cell transplantation LD, lenolidomid, dexamethasone; ECOG, performance status; LDH, lactic dehidrogenase; IPI, International Prognostic Index; PFS, progression-free survival, OS: Overall survival

^a^Median

eNOS *G894T* and *VNTR* genotypes and alleles of the patients were compared statistically with the healthy control group. There was no significant difference between the two groups in terms of *G894T* GG, GT and TT genotypes, and G and T alleles (*p* = 0.201, 0.954, 0.851, 1.000). There was no significant difference between the two groups in terms of *VNTR* BB, BA and AA genotypes, and B and A alleles (*p* = 0.844, 0.496, 0.344, 1.000) (Table 2).

**Table 2:** Genotype and allele frequencies of eNOS variants between patients and controls

*eNOS* G894T	Patients	Controls	OR Exp (B)	95% CI	*p*
	n = 60 (%)	n = 60 (%)			
Genotypes					
GG	4 (6.7)	1 (1.7)	0.227*	0.023–2.207*	0.201*
GT	20 (33.3)	21 (35)	0.977*	0.453–2.111*	0.954*
TT	36 (60)	38 (63.3)	0.883^&^	0.421–1.850^&^	0.851^&^
Alleles					
G	28 (23.3)	23 (19.2)			
T	92 (76.7)	97 (80.8)	1.000^&^	0.550–1.819^&^	1.000^&^
*eNOS* VNTR					
BB	40 (66.7)	42 (70)	0.881^&^	0.406–1.913^&^	0.844^&^
BA	19 (31.6)	15 (25)	0.756*	0.338–1.692*	0.496*
AA	1 (1.7)	3 (5)	3.091*	0.299–31.981*	0.344*
Alleles					
B	99 (82.5)	99 (82.5)	1.000^&^	0.514–1.946^&^	1.000^&^
A	21 (17.5)	21 (17.5)			

^*^OR (95%CI) was adjusted by age and sex

^&^Fisher's Exact Test

In comparison between *G894T* and clinical parameters, aphthous stomatitis was more common in TT genotype, while DMFT > 3 was more common in TG-GG genotype (*p* = 0.035, 0.023) (Table 3). In comparison between *VNTR* genotypes and clinical findings, no statistically significant result was obtained (*p* > 0.05) (Table 4).

**Table 3:** Association between eNOS *G894T* variant and clinical features of patients

*eNOS* G894T	TT	TG-GG	OR Exp(B)	95% CI	*p*
Number of patients	36 (%)	24 (%)			
Males/FEMALES	20/16	11/13			
Age of diagnosis	52 (37–77)	55 (46–81)			
Apthous stomatitis	6 (16.7)	0 (0)	1.200	1.037–1.389	**0.035**^**&**^
DMFT > 2	28 (77.8)	22 (91.2)	0.318	0.061–1.652	0.157^&^
DMFT > 3	18 (50)	19 (79.2)	0.263	0.081–0.858	**0.023**^**&**^

^&^Fisher's Exact Test Pearson Chi-Square ^**&**^Logrank test; ^**@**^OR (95%CI) was adjusted by age and sex; *median; ^**#**^Median test; DMFT: the decayed, missing and filled teeth index. The results that are statistically significant are shown in boldface

**Table 4:** Association between eNOS *VNTR* variant and clinical features of patients

*eNOS* VNTR	BB	BA-AA	OR Exp(B)	95% CI	*p*
Number of patients	40 (%)	20 (%)			
Males/females	19/21	12/8			
Age of diagnosis	54 (37–81)	52 (38–77)			
Apthous stomatitis	5 (12.5)	1 (5)	3.848	0.344–43.043	0.274^@^
DMFT > 2	35 (75)	15 (75)	2.220	0.549–8.980	0.264^@^
DMFT > 3	26 (65)	11 (55)	1.377	0.410–4.622	0.605^@^

^&^Fisher's Exact Test. Pearson Chi-Square ^**&**^Logrank test; ^**@**^ OR (95%CI) was adjusted by age and sex; * median; ^**#**^Median test; DMFT: the decayed, missing and filled teeth index

## Discussion

Considering the frequency of MM and bisphosphonate use, MRONJ emerges as an important clinical problem. To our knowledge, this study is the first study evaluating genetic polymorphisms in MM and MRONJ patients. NO inhibits osteoclast-mediated bone resorption in vitro and in vivo, and its protective effects against bone loss have been demonstrated, especially in postmenopausal women [28]. Decreased NO synthesis enhances osteoclast-mediated bone resorption and increases bone loss [28]. Its effects on angiogenesis, remodeling and osteogenetic activity of bone have been revealed. With its proven effects in both humans and animals, NO constitutes an important working point.

Although our study was conducted on patients using bisphosphonates; bisphosphonates are not the only antiresorptive treatment agent. In a recent meta-analysis [29], the MRONJ effect of denosumab has been proven, and it has even higher risk than zoledronic acid. In the light of all these data, MRONJ does not originate from a single pathologic mechanism; it seems more logical to talk about the unity of factors. MRONJ pathophysiology is basically based on deep inhibition of osteoclast function, which disrupts bone remodeling and creates the clinical picture; however, it does not seem possible to blame osteoclast dysfunction alone. It has been shown that low turnover caused by antiresorptive treatments also increases the risk of infection and creates a cumulative effect [30, 31].

Another important working point is angiogenesis. In a study on pamidronate and zoledronic acid [13], the effects of these two agents on angiogenesis were investigated. In this study, in which zoledronic acid was shown to be a potent angiogenesis inhibitor, it was proven that bisphosphonates inhibit angiogenesis. Although this can be seen as an advantage in preventing metastasis of malignancies, it is an important pathophysiological factor in terms of MRONJ. In this study, in which we aimed to examine the bisphosphonate and these effects, as well as the possible NO polymorphism-related angiogenesis difference and MRONJ relationship, we could not reveal a significant difference with the healthy control group; however, the results obtained with the eNOS *G894T* in terms of aphthous stomatitis and DMFT bring important clinical controversy.

Oral mucosal damage, which is the common point of many studies for MRONJ and seen as a pathophysiology initiating factor, constitutes another important field of study. It has not been clearly demonstrated whether the pathological change mainly begins in the bone or is caused by mucosal damage. We see that bisphosphonates and mucosal damage have been demonstrated in studies conducted. The toxicity of bisphosphonates on mucosal cells has been documented previously [14, 32, 33]. Epithelial toxicity of bisphosphonates has been demonstrated using Caco-2 cells in two different important studies. Caco-2 cells are cells derived from gastric adenocarcinoma, similar to the gastric epithelium. In the study conducted by Twiss et al. [32], the toxicity of pamidronate and alendronate to epithelial cells has been proven. In the presence of calcium, pamidronate and alendronate have been found toxic to cells and pamidronate is more toxic than alendronate. Olpadronate caused toxicity at concentrations ten times higher than the toxic concentrations of pamidronate. In the absence of calcium, precise signs of toxicity were observed with pamidronate only at clinically relevant concentrations. The most toxic bisphosphonate was found to be pamidronate. In the study of Suri et al. [33] bisphosphonates appear to cause inhibition of the apoptosis of epithelial cells and/or proliferation of undifferentiated epithelial cells (due to loss of prenylated proteins and sterols) and may contribute to the esophagitis and ulcer in the gastrointestinal tract.

In terms of eNOS polymorphism, which is a significant statistical finding of our study, we see that similar data in the literature are related to Behçet's disease, where recurrent aphthous stomatitis is also common. There was a significant association between recurrent aphthous stomatitis and inheritance of single-nucleotide polymorphism *rs2297518* (*p* = 0.006) [34]. In another study, no relationship was found between the studied eNOS polymorphisms and aphthous stomatitis [35].

In the light of all these effects, it can be said that bisphosphonates show toxicity to the mucosa apart from their effects on MRONJ pathology. Our study includes the first results in the literature in terms of mucosal damage seen as the pathophysiological mechanism that initiates MRONJ. For eNOS *G894T* polymorphism, significantly more aphthous stomatitis was observed in TT genotype, while having a DMFT > 3 score in TG-GG genotype was found to be significantly higher. It is thought that there will be genotype differences between MRONJ patients and the healthy population in the evaluation to be made with a larger patient population.

Synthesized by eNOS, NO plays an important role in blood flow regulation as a potent vasodilator, anti-thrombotic, anti-inflammatory and anti-proliferative. The eNOS polymorphism plays an important role in endothelial dysfunction [36]. The eNOS *G894T* variant plays a role in the pathogenesis of a variety of diseases such as cardiovascular disease and erectile dysfunction [37, 38]. However, we see that various studies have been conducted on ischemic stroke. Regarding the relationship between the eNOS *G894T* polymorphism and the risk of ischemic stroke, the results of the study of Diakite et al. [39] show that GT and TT genotypes are significantly associated with ischemic stroke. These data are supported by two separate studies [40, 41]. Considering that NOS polymorphism and the resulting endothelial dysfunction may also play a role in MRONJ pathophysiology, it should be said that our study results point to a very important and new area. In parallel with the publications related to ischemic storm and atherosclerosis, DMFT score was found above 3 in GT and TT genotypes in our study. It should be emphasized that different eNOS polymorphisms and associated endothelial dysfunction are the first study results to be associated with MRONJ.

It is necessary to summarize our study results as follows and include them in the literature: eNOS induces osteogenesis in bone metabolism, with its regulatory effects on bone remodeling, and also NO induced angiogenesis takes place indirectly with its protective effect on endothelial functions. We see that these polymorphisms affecting the entire process of bone remodeling and angiogenesis, especially mucosal damage, which is the triggering factor of MRONJ pathology, have been revealed in the MM patient group.

The most important limitation of our study is that our patient and control groups are small. For this reason, it can be thought that there is no significant difference in genotypes between MRONJ patients and the control group. In addition, although the use of only zoledronic acid in the patient population can be seen as a statistical advantage, it can be seen as an obstacle to the evaluation of possible polymorphism differences that will occur in MRONJ cases that occur with different treatment subtypes. With more meaningful results to be achieved in a larger patient population, NO-based new approaches in MRONJ treatment may come into question. In addition, the polymorphisms we studied need to be examined in cases of bisphosphonates or similar antiresorptive therapy unrelated osteonecrosis. Additional studies are needed to say that the polymorphisms related to our results are specific to the use of bisphosphonates or MM patients only.

## Conclusion

In conclusion, to our best of knowledge, our study is the first in the MM patient group in terms of MRONJ and eNOS polymorphism features. In comparison between eNOS *G894T* and clinical parameters, aphthous stomatitis was seen more in TT genotype, while DMFT was more common in TG-GG genotype > 3; Considering the MRONJ initiating factors, it is necessary to emphasize the importance of our study results. It should be seen as an important step for new studies towards MRONJ and its treatment.

## Data Availability

The authors declare that data supporting the findings of this study are available within the referenced articles.

## Abbreviations

MM: Multiple myeloma
PET/CT: Positron emission tomography/computed tomography
MR: Magnetic resonance
MRONJ: Medication-related osteonecrosis of the jaw
CT: Computed tomography
RANKL: Nuclear-KB ligand receptor
DKK: Dickkopf-1
NOS: Nitric oxide synthase
OS: Overall survival
PFS: Progression free survival
DMFT: Decayed-missing-filled teeth index
